# Annotating and prioritizing genomic variants using the Ensembl Variant Effect Predictor—A tutorial

**DOI:** 10.1002/humu.24298

**Published:** 2021-12-02

**Authors:** Sarah E. Hunt, Benjamin Moore, Ridwan M. Amode, Irina M. Armean, Diana Lemos, Aleena Mushtaq, Andrew Parton, Helen Schuilenburg, Michał Szpak, Anja Thormann, Emily Perry, Stephen J. Trevanion, Paul Flicek, Andrew D. Yates, Fiona Cunningham

**Affiliations:** European Molecular Biology Laboratory, European Bioinformatics Institute, Wellcome Genome Campus, Hinxton, Cambridge, UK

**Keywords:** “molecular consequence”, filtering, variant annotation, variant prioritisation, VEP

## Abstract

The Ensembl Variant Effect Predictor (VEP) is a freely available, open-source tool for the annotation and filtering of genomic variants. It predicts variant molecular consequences using the Ensembl/GENCODE or RefSeq gene sets. It also reports phenotype associations from databases such as ClinVar, allele frequencies from studies including gnomAD, and predictions of deleteriousness from tools such as Sorting Intolerant From Tolerant and Combined Annotation Dependent Depletion. Ensembl VEP includes filtering options to customize variant prioritization. It is well supported and updated roughly quarterly to incorporate the latest gene, variant, and phenotype association information. Ensembl VEP analysis can be performed using a highly configurable, extensible command-line tool, a Representational State Transfer application programming interface, and a user-friendly web interface. These access methods are designed to suit different levels of bioinformatics experience and meet different needs in terms of data size, visualization, and flexibility. In this tutorial, we will describe performing variant annotation using the Ensembl VEP web tool, which enables sophisticated analysis through a simple interface.

## 1 Introduction

Genome and exome sequencing are becoming routine in clinical research and diagnostic settings, as an individual’s genotype may provide insight into disease mechanism, progression, and treatment. Each sequenced genome contains 4.1–5.0 million variant sites (1000 Genomes Project Consortium et al., 2015), many of which will be rare but benign alleles, so additional information is required to enable variant interpretation and prioritization. As the scale of data production increases, robust and efficient software tools are needed to support variant annotation and filtering.

Variant interpretation requires (i) the mapping of variants to transcripts and predictions of molecular consequence; (ii) the consideration of all current knowledge relating to a variant; and (iii) the application of predictive algorithms to evaluate the impact of change at the locus. Appropriate resources are now available to facilitate variant interpretation and include: reference gene sets that are regularly updated; assertions of genotype-phenotype association continue to grow in the key databases and literature; population frequency studies that are expanding to include more individuals and report more detailed catalogs of rare variants, and variant pathogenicity prediction, which is an active area of tool development.

In the Ensembl Project (Howe et al., 2021) we create high-quality gene sets, predict genomic regions involved in gene regulation, and collate large-scale sets of variant and phenotype association data. Ensembl VEP (McLaren et al., 2016) builds on these resources and integrates results from variant assessment algorithms to enable convenient but extensive variant annotation. We provide regular updates, approximately every 3 months, to both the VEP software and associated data to ensure the latest information can be used for analysis. Here we present a tutorial describing the Ensembl VEP web interface, detailing the available analyses options and filters.

## 2 Data Input

**Figure 1:**
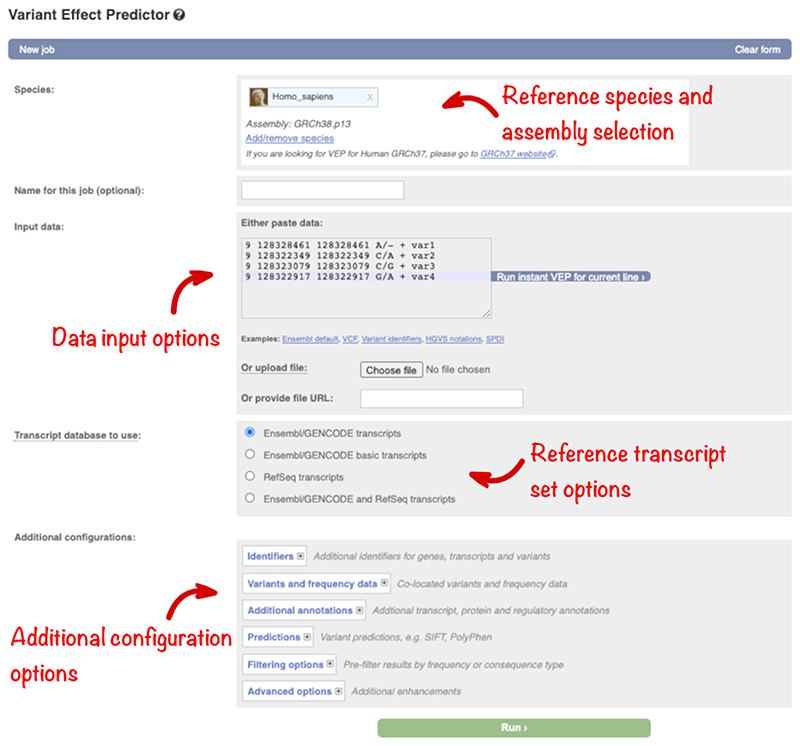
The Ensembl VEP web interface showing species/assembly selection, data input, transcript set selection, and additional groups of configuration options

Navigate to the Ensembl VEP homepage by clicking on the “VEP” link in the blue navigation bar on the Ensembl homepage (https://www.ensembl.org/index.html). The Ensembl VEP homepage links to the three different VEP interfaces and detailed documentation. Click on “Launch VEP” to open the web form, which is divided into sections for data input and optional analysis configuration (Figure 1).

The human GRCh38 assembly is selected by default, but a link provides access to a GRCh37 dedicated tool. To make the management of multiple analyses simpler, a name can be assigned to each job.

Data can be input by (1) pasting into the text box, (2) uploading a file, or (3) by providing a URL for a file on a public server. The text box is suitable for small-scale datasets. To analyze a larger data set, provide a URL or use the file upload option which supports a maximum file size of 50 megabytes (or around two million lines in a compressed variant call format [VCF] file).

Ensembl VEP supports a range of data input formats including;

- VCF;
- Human Genome Variation Society (HGVS) descriptions (den Dunnen et al., 2016), using Ensembl, RefSeq or Locus Reference Genomic (LRG) accessions;
- Variant identifiers from databases including dbSNP (Sherry, 2001), ClinVar (Landrum et al., 2018), and UniProt (The UniProt Consortium et al., 2021);
- Ambiguous gene-based descriptions often used in literature (e.g., “*BRCA2*:p.Val2466Ala”).

VCF is the standard exchange format used in next-generation sequencing pipelines so Ensembl VEP is optimized to analyze variants in this format.

Further options for selecting the reference transcript set as well as retrieving additional annotations including related identifiers, allele frequencies, pathogenicity predictions, and phenotype annotations can be found in the expandable panels and will be explored in more detail below.

## 3 Transcript Set Selection

Predicting the molecular consequence of a genomic variant is an essential step in interpretation and requires extensive, accurate gene annotation. There are two commonly used human gene sets: Ensembl/GENCODE (Frankish et al., 2021) and RefSeq (O’Leary et al., 2016). Both sets are generated using similar but slightly different evidence and algorithms, and so differ slightly. VEP can analyze variants using either gene set, or the combined group, or GENCODE Basic (which contains a small subset of representative transcripts for each gene). Select your preference in the “Transcript database to use” section (Figure 1).

The Ensembl VEP algorithm compares each variant to each transcript in the selected set and reports the relative transcript location of the variant (e.g., exonic, upstream) with any predicted molecular consequence (e.g., missense, frameshift). Consequences are described using Sequence Ontology terms (SO; Cunningham et al., 2015) to enable comparison and integration with results from other systems.

### 3.1 Transcript-related identifiers

**Figure 2:**
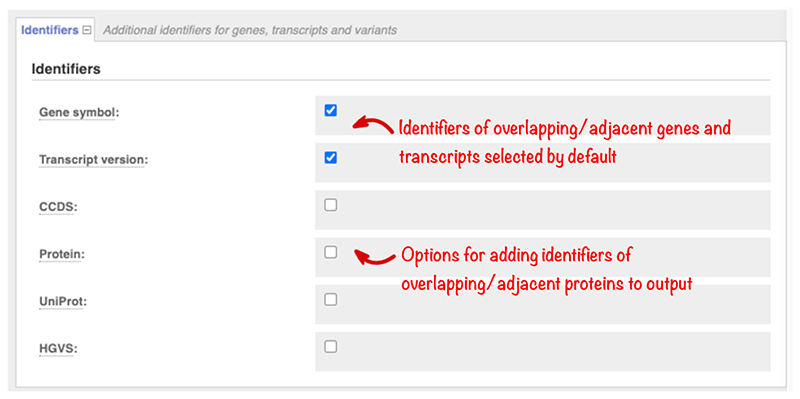
The “Identifiers” section, which allows the selection of gene, protein, and HGVS identifiers

Gene symbols assigned by the HUGO (Human Genome Organisation) Gene Nomenclature Committee (HGNC), versioned transcript accessions, and transcript types (e.g., *AGT*, ENST00000366667.6, protein-coding, respectively) are returned by default. Use the “Identifiers” section (Figure 2) to add further information, including Ensembl or RefSeq protein identifiers, UniProt protein accessions, and HGVS variant descriptions at protein and transcript level to your output.

### 3.2 Frequencies and citations

With over seven hundred million variants in dbSNP (version 154, May 2020) alone, the majority of variants found in an individual will have already been described. This information can be crucial to interpretation. Ensembl VEP searches records from databases including dbSNP, the Catalogue Of Somatic Mutations In Cancer (COSMIC), and the Human Gene Mutation Database (HGMD) and reports any variants at the same location as your input variants. For databases with redistribution restrictions, variants are matched on location alone (i.e., with no allele specificity) and names are reported. For fully open databases, variants are matched by allele, and key additional information is reported. By default, we only report matches to variants passing our quality filtering (e.g., those mapping to multiple genomic locations are excluded); to include all variants in the search check the “Include flagged variants” option.

**Figure 3:**
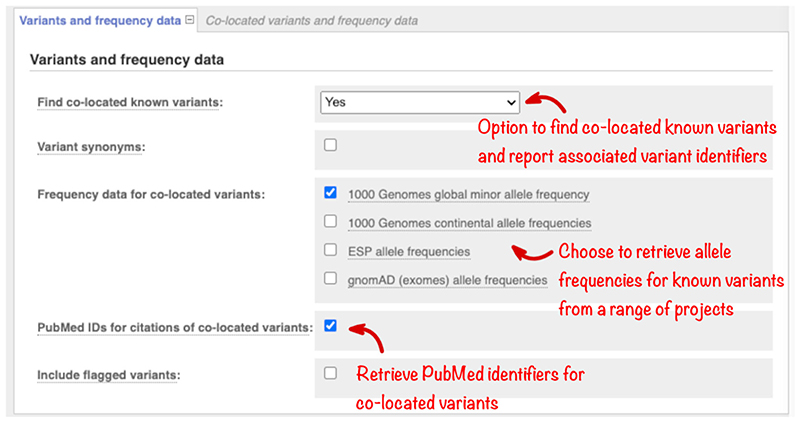
The “Variants and frequency data” section, which allows the selection of information known about variants at the same location

In rare disease studies, it is useful to filter out variants using reference population frequencies, as variants common in the general population are less likely to be causative. Use the “Variants and frequency data” section (Figure 3) to select the reference data set to be searched. Allele frequencies from the Genome Aggregation Database (gnomAD; Karczewski et al., 2020) and 1000 Genomes Project (1000 Genomes Project Consortium et al., 2015) are currently available.

The American College of Medical Genetics and Genomics (ACMG) guidelines (Richards et al., 2015) use 5% allele frequency as stand-alone evidence a variant allele is not pathogenic. For a single causative variant, ACMG recommends frequency filters should be selected to be higher than disease prevalence. Filter cut-offs should be higher if it is possible multiple variants are acting together.

Select the “Variant synonyms” option to display the names of variants in databases such as ClinVar, UniProt, and PharmGKB. In your results, the names will be linked to the relevant entries in the source databases, so the details held in these resources can be examined. Check the “PubMed identifiers” button to return a list of any publications describing the variant with links to full-text resources where available. Variant citations are imported from a number of sources including manually curated records. These can contain occasional incorrect results which cannot be filtered out computationally, though errors are usually obvious on review. Citation and synonym information is matched on variant name or location and is not allele-specific.

### 3.3 Transcript selection

**Figure 4:**
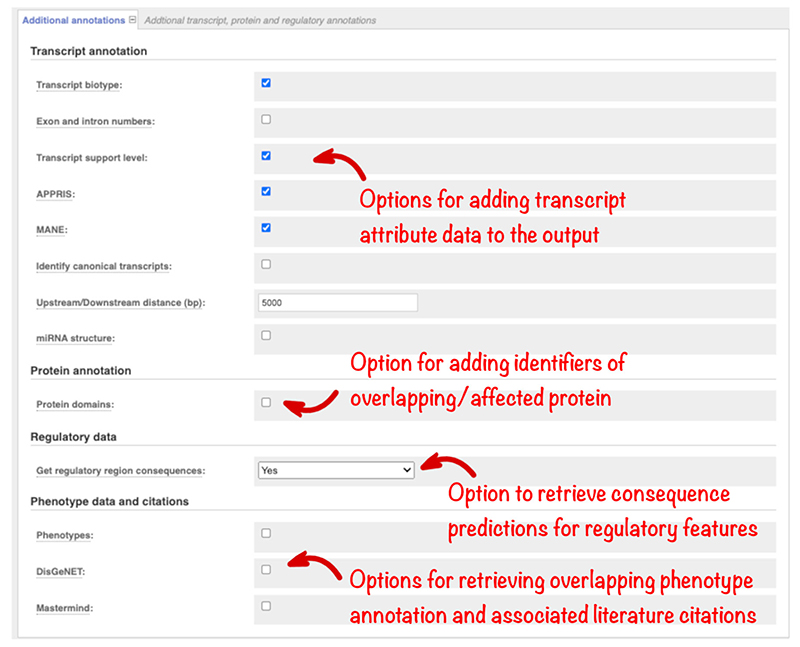
The “Additional annotations” section, which allows the selection of transcript, protein domain, regulatory region, and phenotype annotations

Transcriptomic sequencing from multiple tissues has resulted in the annotation of increasing numbers of transcript isoforms for many genes. Assessing large numbers of predictions for each variant is time-consuming but important to ensure no information is missed. To support downstream filtering VEP reports transcript type (such as protein-coding or pseudogene) and, for Ensembl transcripts, two prioritization metrics. Transcript Support Level (TSL) summarises the amount of evidence supporting a transcript into a numeric score. APPRIS (Rodriguez et al., 2018) identifies principal transcript isoforms for genes in vertebrate species using protein structural information, functionally important residues, and evidence from cross-species alignments. These options are listed in the “Transcript annotation” section and are reported in Ensembl VEP results by default (Figure 4).

MANE (Matched Annotation from NCBI and EMBL-EBI) transcripts are also reported by default to facilitate transcript prioritization. MANE Select transcripts are single representative transcripts for each protein-coding human gene, chosen by the European Molecular Biology Laboratory’s European Bioinformatics Institute (EMBL-EBI) and the National Center for Biotechnology Information (NCBI). They are recommended as the default transcript where one is needed for reporting. An additional transcript is required to report all clinically relevant variants in a small number of genes, including *LAMA3* and *SCN2A*. MANE Plus Clinical transcripts are being assigned to meet this need. MANE transcripts are identical between the RefSeq and Ensembl/GENCODE sets and match the GRCh38 reference genome sequence. MANE Select transcripts are available for 78% of proteincoding genes and MANE Plus Clinical transcripts for 55 genes in Ensembl release 104 (May 2021). Selection of the MANE option flags these recommended transcripts and reports both RefSeq and Ensembl transcript identifiers.

The Ensembl canonical transcript is a single default transcript available for every gene, in every species. The same Ensembl algorithm is used to pick MANE Select transcript and the canonical transcript in humans, so the two are the same where a MANE Select exists. Check the “Identify canonical transcripts” option to highlight these transcripts in your results if you require a default for every gene.

Additional transcript configuration options are available in the ‘Transcript annotation’ section (Figure 4). The distance upstream and downstream of a transcript in which variants are reported can be changed from the default of 5 kb, which is useful to reduce the number of variant annotations returned if these regions are not relevant in an analysis. For variants falling in predicted microRNA (miRNA), it is also useful to know where the variant lies in the secondary structure. This is reported using the miRNA structure option.

### 3.4 Protein domains

When a variant maps to the protein, understanding which domain it falls in can provide clues as to its possible impact on function. InterPro is an integrated resource for protein families, domains, and sites, combining information from several different protein signature databases (Blum et al., 2021). We run InterProScan (Jones et al., 2014) on all Ensembl protein sequences to identify domains, and these are reported in VEP. Check the “Protein domains” option (Figure 4) to report these results and any overlapping Protein Data Bank in Europe (PDBe, Armstrong, et al., 2019) structures.

### 3.5 Regulatory elements

Variants in the noncoding regions of the genome are more difficult to interpret than those falling within genes and are also important in disease (Zhang & Lupski, 2015). In the Ensembl project, we use data from large-scale projects including Encyclopedia of DNA Elements (ENCODE), the International Human Epigenome Consortium (IHEC), and Blueprint, to predict regions in the human genome that influence gene regulation. We classify them into types such as “promoter” and “enhancer” (Zerbino et al., 2015). Select the “Get regulatory region consequences” option (Figure 4) to identify where your variants overlap such regions. This analysis can be configured to report all results or only those from specific cell types.

### 3.6 Phenotype and disease associations

Access to phenotype or disease associations previously reported for your variants or the genes they overlap is essential. There is a large body of information available in different databases but performing multiple searches across different resources is timeconsuming. In Ensembl, we aggregate phenotype and disease associations from a variety of sources, including Orphanet (an online rare disease and orphan drug database. © INSERM 1999. Available on http://www.orpha.net), the COSMIC Cancer Gene Census (Sondka et al., 2018), ClinVar and the National Human Genome Research Institute-European Bioinformatics Institute Genome-wide association study Catalog (NHGRI-EBI GWAS Catalog; Buniello et al., 2019), into a standardized format (Hunt et al., 2018). This information is searched by Ensembl VEP and summary information reported. ClinVar assertions of variant clinical significance are reported by default and, importantly, these are matched by allele and not just variant location. Select the “Phenotypes” option (Figure 4) to retrieve a list of phenotype associations for overlapping genes and previously reported variants, with links to fuller information.

Results from additional sources are available. DisGeNET (Piñero et al., 2019) is a database of gene and variant disease associations. Select this option to view summary results including disease names and PubMed identifiers, which are linked to full-text publications. The Mastermind Genomic Search Engine (Chunn et al., 2020) (https://www.genomenon.com/mastermind) holds gene, variant, disease, phenotype, and therapy evidence mined from millions of scientific articles. Select this option to return links to the Mastermind website, which is free to access, with registration.

### 3.7 Prediction packages

An increasing number of pathogenicity scoring algorithms are being developed to aid variant interpretation. It must, however, be remembered that predictions often use the same training sets and/or evidence so agreement between two algorithms does not necessarily provide additional evidence for a rating. We calculate scores for all possible amino acid substitutions in all Ensembl proteins using SIFT (Kumar et al., 2009) and PolyPhen-2 (Adzhubei et al., 2010). These results are returned by default.

**Figure 5:**
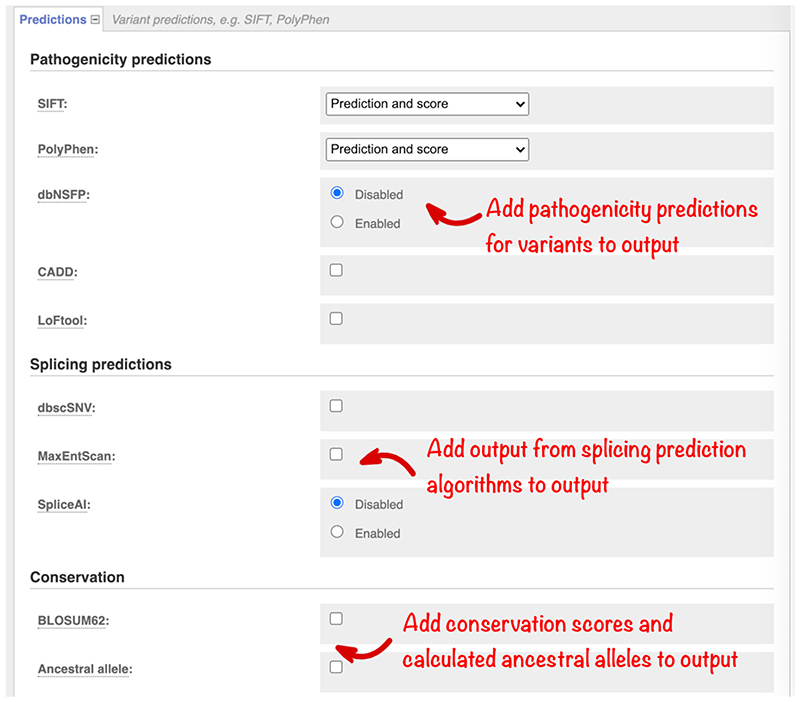
The “Predictions” section, which allows the selection of different pathogenicity, splicing, and conservation predictions

dbNSFP, the database for nonsynonymous SNPs’ functional predictions (Liu et al., 2020) contains pre-calculated scores for over 20 algorithms. Select this option (Figure 5), to browse the “Fields to include” menu and configure the precise results set to be returned. CADD (Rentzsch et al., 2019) is a framework for scoring the deleteriousness of genomic variants using a wide range of different information including conservation, functional information, and protein level pathogenicity predictions. Select this option to view scores for variants in both coding and noncoding loci.

Variants that disrupt splicing have also been implicated in human disease (Ward & Cooper, 2010). We optionally report results from the well-established MaxEntScan (Yeo & Burge, 2004); SpliceAI (Jaganathan et al., 2019), which takes a machine learning approach; and the ensemble scores provided in the dbscSNV (Liu et al., 2020) database. Select these options in the “Splicing predictions” section (Figure 5).

Considering sequence constraint and conservation can help interpret how well a sequence change at a particular location may be tolerated. Catalogs of variants in dense population samples have enabled improved estimation of selection against changes that result in loss of function for a gene. Scores from one such algorithm, LoFtool (Fadista et al., 2016), are available. We also optionally report BLOSUM62 (Henikoff & Henikoff, 1992) scores for missense variants as a classic measure of the impact of changing one amino acid for another. In Ensembl, we infer genome-wide ancestral sequences (Paten et al., 2008) for different groups of species. Select the “Ancestral Allele” option (Figure 5) to obtain the ancestral allele predicted from the alignment of 12 primate species, including homo sapiens.

### 3.8 Filtering and advanced options

**Figure 6:**
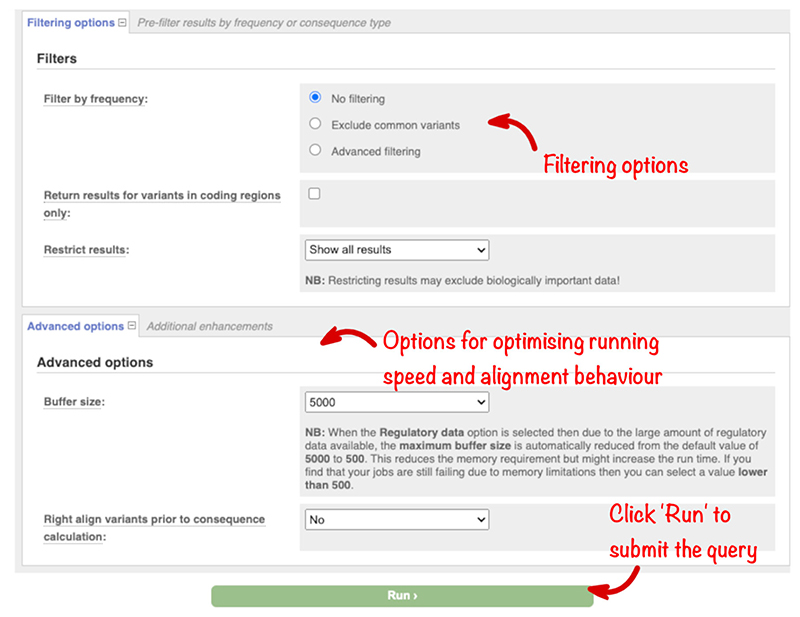
Filtering and advanced options

The options in these sections will not be required for the majority of analyses. The “Filters” section (Figure 6) allows the results returned to be restricted by allele frequency, to contain only variants in the coding sequence, or to be reduced to a subset of the available variant-transcript combinations. However, we recommend instead filtering results after the analysis, which allows for greater flexibility. The “Advanced options” allow you to change the way VEP analyses variants internally (a smaller batch size will reduce memory requirements but increase run time) and control whether insertion and deletions in repetitive sequence are expressed at their most 3’ position before consequence evaluation.

## 4 Results

Having configured your analysis, click the “Run” button at the bottom of the form. Analysis jobs run on our compute farm and the time required will depend on the number of input variants and range of options chosen. The “Recent jobs” table displays the status of all your analyses and has options to edit and resubmit, share or discard jobs. Results can be saved by logging into an Ensembl account. Once a job has the status of “Done,” clicking on “View Results” will display the results table.

**Figure 7:**
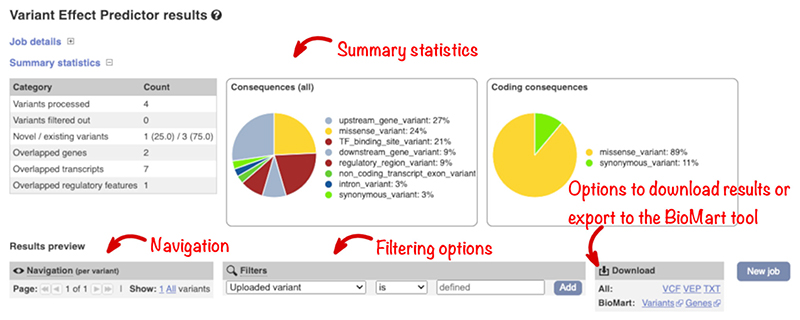
The results page with summary statistics and options for filtering and downloading the results table

Summary statistics and charts display an overview of the results on the output page (Figure 7). There is also a table with a preview of the detailed results and a simple interface to configure filtering of the output. To aid variant prioritization, multiple filters can be combined using basic logical relationships, allowing the creation of complex customized queries. For example “Consequence is protein_altering_variant” plus “CADD PHRED >=30” plus “gnomAD AF is not defined” will report variants which are predicted to change protein sequence, are in the 0.1% most deleterious changes predicted by CADD and are not seen in the gnomAD exome variant set. Importantly, we report the most specific SO term but enable querying by parent terms. For example, when the consequence of “protein-altering variant” is selected, missense and frameshift variants are reported.

The results interface allows you to download your output in VCF and other formats for further analysis or export the variation or gene list to the Ensembl BioMart tool to extract additional data, such as gene homologs and sequences.

**Figure 8:**
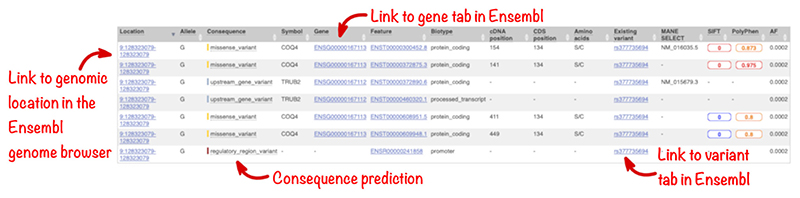
The results table showing predicted molecular consequences and links to the location and overlapping genes and variant displays within the Ensembl genome browser

Results are displayed in a table (Figure 8) with a single line per combination of variant allele and transcript or regulatory element. Click on the “Show/hide columns” button to configure which columns are displayed if you wish to view a subset of the results. Cells containing many records (as can happen, e.g., for PubMed IDs) will initially be compressed and need expanding to view. The results table displays only a summary of the information available for a variant. You can easily examine the evidence for your variants of interest in greater detail. Links enable you to access relevant publications in Europe PMC or view details in resources such as UniProt, ClinVar, and PDBe. The table is also a convenient access point to data held in Ensembl: it has links to the variant location on the genome browser and detailed information about any genes, transcripts, or variants the input variant overlaps.

### 4.1 Structural variants

Ensembl VEP is currently optimized for the annotation of short variants, however basic annotation of structural variants (SVs), with defined boundaries within a chromosome, is supported. SVs should be input using VCF format and the length of the variant must be derivable from either “END” or “SVLEN” keys. All transcripts an SV overlaps are reported and SO consequence terms are assigned to report whether the variant results in the deletion or duplication of part or all of the transcript. Due to the longer genomic regions involved, analysis of SVs is slower and more memory-intensive than for short variants and it is advisable to reduce buffer size using the “Advanced Options” for more efficient analysis.

## 5 Ensembl VEP Interfaces

The Ensembl VEP web tool enables analysis configuration and results filtering via a simple interface. It is ideal for analyzing small sets of variants and interactively assessing the results. We provide two other interfaces that are more appropriate for the integration of VEP annotations in web views or for large-scale analyses. Here we briefly describe these REST and command-line interfaces.

Language-agnostic computational access to VEP analysis is available through the Ensembl REST API. The VEP REST service (https://rest.ensembl.org) supports similar options to the web tool and is suitable for programmatic integration into web pages or analysis pipelines. HGVS notation, position, and allele-based descriptions and a range of common variant names are supported as input and up to 200 variants can be submitted in a single request.

The command-line tool is the most powerful and flexible way to use Ensembl VEP. It supports more analysis options than the other interfaces. There is also no limit on input file size, making it suitable for the annotation of large variant sets identified through whole-genome sequencing. The use of custom gene, variant, and other annotation sets is supported, enabling analysis against private data. While VEP can be run by anyone comfortable with command-line tools, those with basic programming skills can simply create extensions to add novel, custom functionality. Run time depends on the number and complexity of options selected: basic analysis of a whole exome (~200,000 variants) takes under 5 min while a single genome (~4.5 million variants) will take around an hour. A Docker image is available to simplify installation. A results-filtering tool is also available in the Ensembl VEP command-line package. Full instructions for installation and options for running Ensembl VEP locally can be found in our online documentation (https://www.ensembl.org/vep).

## 6 Case Study

A region of chromosome 11 has been sequenced for a number of samples. A VCF file containing information about the identified variants has been produced through a process of alignment and variant calling using the GRCh38 reference genome assembly. Ensembl VEP has been used to annotate the variants with information about allele frequencies reported in the 1000 Genomes project and phenotype annotations. The input VCF file and the output files in VCF and VEP format can be found in the supplementary materials.

To produce these output files, run the web-based VEP tool using the provided input data with default settings as well as selecting the options to retrieve the 1000 Genomes continental allele frequencies and overlapping phenotype annotations.

When viewing the Ensembl VEP output in the web browser, the summary table shows that the data set contains 15 variants that overlap three genes and one regulatory feature.

In the full results table, the first set of columns reports information about the variants and the features they overlap. Where the feature is a transcript, you will find the gene symbol and stable ID and the transcript stable ID and biotype. In this data set, all variants overlap the transcripts of the HBB gene.

The predicted effects on transcripts can be found in subsequent columns, including the position of the variant in terms of the exon number, cDNA, CDS and protein, the amino acid and codon change, as well as transcript flags, such as MANE, which can be used in variant prioritization and reporting, and pathogenicity scores. The pathogenicity scores are shown as numbers with colored highlights to indicate the prediction.

Where the variant is known, its identifier is listed in the “Existing variant” column, with a link out to the variant page within Ensembl. In this example, identifiers from dbSNP, COSMIC, and/or HGMD can be found for each variant.

By default, Ensembl VEP also reports the 1000 Genomes project global allele frequency (AF in the table). In our query, we also selected the option to retrieve allele frequencies from the 1000 Genomes continental populations and these data are displayed in separate columns.

To illustrate filtering, we will look for variants that are not rare in a 1000 Genomes population but do have associations with disease reported in ClinVar. Find variants that are observed at a frequency of 5% or greater in the African continental population of the 1000 Genomes project by selecting a filter of “AFR AF >0.05” and clicking “Add.” Find variants with information in ClinVar by entering a filter of ‘Clinical Significance is defined’ and clicking “Add.”

**Figure 9:**
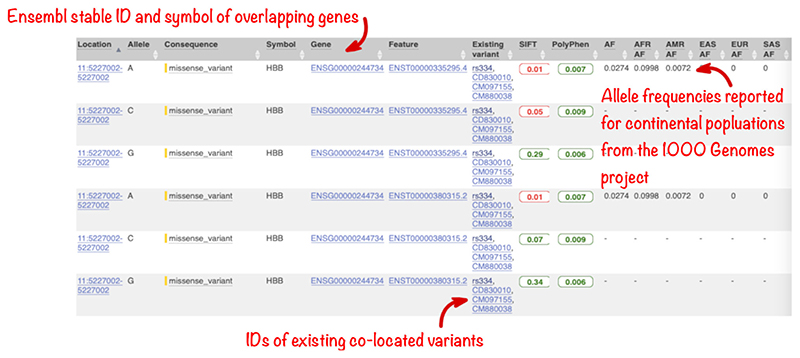
Results table for example input VCF file showing predicted molecular consequences and links to the location, gene, and variant tabs within the Ensembl genome browser for overlapping features as well as SIFT and PolyPhen-2 predictions and allele frequencies for continental populations for the 1000 Genomes project. VCF, variant call format

**Figure 10:**
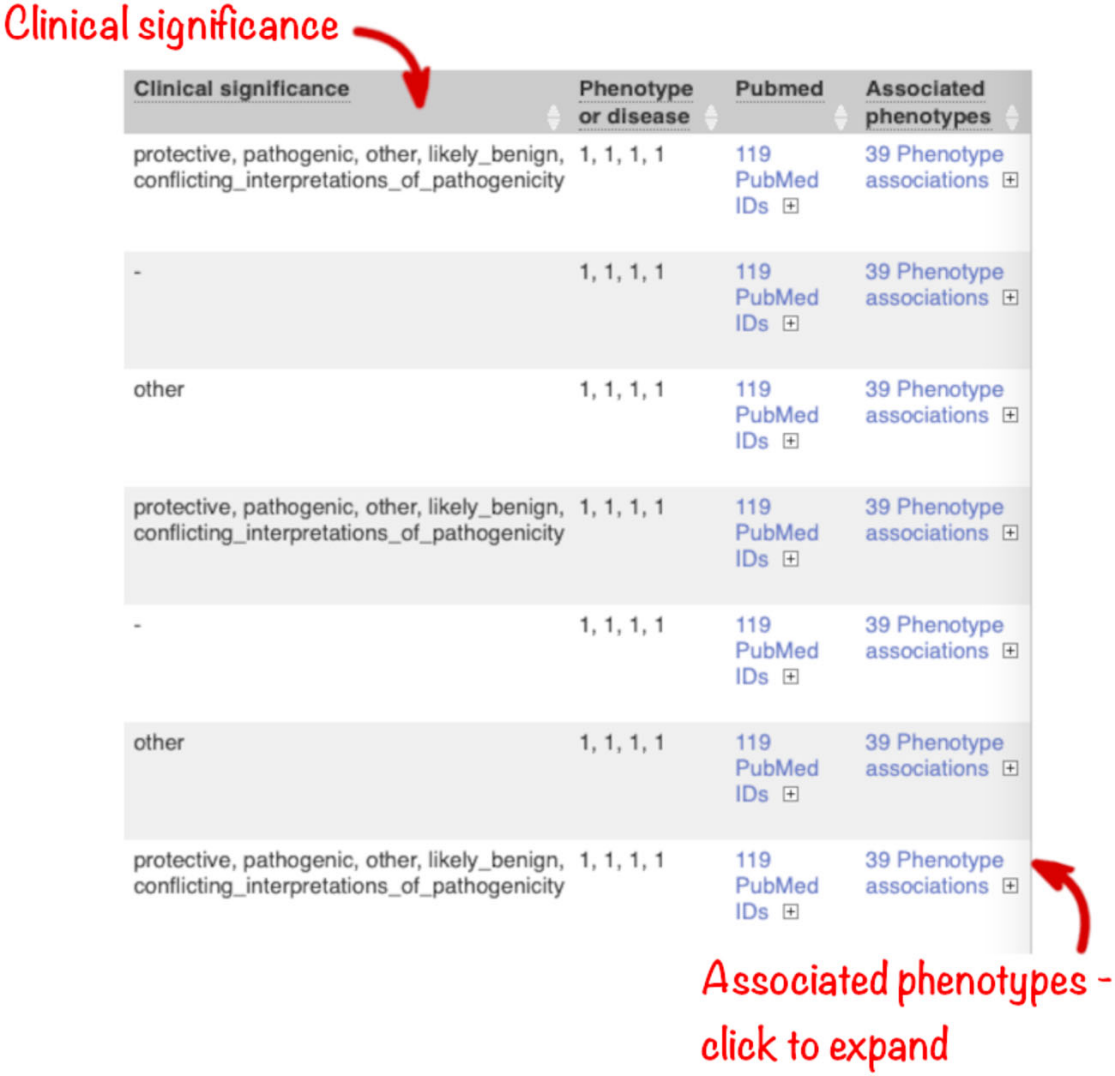
Results table for example input VCF file showing clinical significance, associated PubMed IDs, and associated phenotypes. VCF, variant call format

In this query, the variant with ID rs334 has an alternative allele frequency of 0.0998 in the African continental population, 0.0072 in the American continental population, but 0 in the East Asian, South Asian, and European continental populations (Figure 9). In subsequent columns, the ClinVar clinical significance and the phenotypes associated with known variants or with the genes affected by the variants are reported. In this query, the variant with ID rs334 is described as both protective and pathogenic as well as being associated with 39 phenotypes including malaria, anemia, and beta-thalassemia (Figure 10).

To export the Ensembl VEP output as a VCF file, select VCF for the Download interface in the results table header. When exported as VCF, the VEP annotation is listed under CSQ in the INFO column. The “VEP” and “TXT” format options have multiple output lines for each variant. Each shows the predicted impact of a single variant allele on a single transcript or regulatory feature, with all relevant annotations.

## 7 Conclusion

The Ensembl VEP web tool enables the flexible configuration of variant analysis from an extensive range of options via a simple interface. It allows customizable filtering so you can interrogate and understand your results. It links out to detailed resources, both within the Ensembl browser and other key websites. The regular updating of the reference data and analysis tools supported within Ensembl VEP makes it an essential tool for variant annotation, filtering, and prioritization.

## Supplementary Material

Input file

Output file VCF

Output file VEP

## Data Availability

No new data were created or analyzed in this study. Publicly available data is integrated into the Ensembl variation resources. Reference data packaged for use in Ensembl VEP is available from our FTP site in release-specific directories for example: http://ftp.ensembl.org/pub/release-103/variation/vep/.
